# Modeling of subglacial hydrological development following rapid supraglacial lake drainage

**DOI:** 10.1002/2014JF003333

**Published:** 2015-06-30

**Authors:** C F Dow, B Kulessa, I C Rutt, V C Tsai, S Pimentel, S H Doyle, D van As, K Lindbäck, R Pettersson, G A Jones, A Hubbard

**Affiliations:** 1 Glaciology Group, College of Science, Swansea UniversitySwansea, UK; 2 Cryospheric Sciences Laboratory, NASA Goddard Space Flight CenterGreenbelt, Maryland, USA; 3 Seismological Laboratory, California Institute of TechnologyPasadena, California, USA; 4 Faculty of Natural and Applied Sciences, Department of Mathematics, Trinity Western UniversityLangley, British Columbia, Canada; 5 Institute of Geography and Earth Sciences, Aberystwyth UniversityAberystwyth, UK; 6 Geological Survey of Denmark and GreenlandCopenhagen, Denmark; 7 Department of Earth Sciences, Uppsala UniversityUppsala, Sweden

**Keywords:** Greenland ice sheet, subglacial hydrology, lake drainage, modeling

## Abstract

The rapid drainage of supraglacial lakes injects substantial volumes of water to the bed of the Greenland ice sheet over short timescales. The effect of these water pulses on the development of basal hydrological systems is largely unknown. To address this, we develop a lake drainage model incorporating both (1) a subglacial radial flux element driven by elastic hydraulic jacking and (2) downstream drainage through a linked channelized and distributed system. Here we present the model and examine whether substantial, efficient subglacial channels can form during or following lake drainage events and their effect on the water pressure in the surrounding distributed system. We force the model with field data from a lake drainage site, 70 km from the terminus of Russell Glacier in West Greenland. The model outputs suggest that efficient subglacial channels do not readily form in the vicinity of the lake during rapid drainage and instead water is evacuated primarily by a transient turbulent sheet and the distributed system. Following lake drainage, channels grow but are not large enough to reduce the water pressure in the surrounding distributed system, unless preexisting channels are present throughout the domain. Our results have implications for the analysis of subglacial hydrological systems in regions where rapid lake drainage provides the primary mechanism for surface-to-bed connections.

**Key Points:**

- Model for subglacial hydrological analysis of rapid lake drainage events
- Limited subglacial channel growth during and following rapid lake drainage
- Persistence of distributed drainage in inland areas where channel growth is limited

## 1. Introduction

The role played by basal water in the dynamics of the Greenland ice sheet (GrIS) is of considerable contemporary interest in glaciology. It has been argued that Greenlandic subglacial drainage behaves in a similar way to that observed in alpine valley glaciers, where a “spring event” causes ice acceleration above the mean winter velocity. This is followed by the growth of efficient channels that results in deceleration of ice flow as the melt season progresses [*Bartholomew et al.*, 2010, 2011b; *Cowton et al.*, 2013; *Joughin et al.*, 2013; *Sole et al.*, 2013; *van de Wal et al.*, 2015]. It is becoming increasingly clear, however, that the system is more complex than suggested by this general model. Global positioning system (GPS) measurements of surface ice motion reveal that the seasonal velocity cycle is punctuated by transient acceleration events [e.g., *Hoffman et al.*, 2011], which are argued to occur when water inputs exceed the capacity of the subglacial channels [e.g., *Bartholomew et al.*, 2012]. In addition, recent work by *Andrews et al.* [2014] suggests that it is not just the size of channels that influences regional drainage systems but the connectivity of channels with the surrounding distributed system, which allows lower pressure channels to draw water from the higher pressure distributed system, thus causing ice deceleration.

On alpine glaciers, ice typically accelerates in response to upstream migration of the snowline and consequent meltwater access to the bed through moulins [*Nienow et al.*, 1998]. In Greenland, this is complicated by the storage of large volumes of water in supraglacial lakes, which either overtop and drain into downstream moulins, or rapidly drain in situ [*Das et al.*, 2008; *Selmes et al.*, 2011; *Liang et al.*, 2012; *Doyle et al.*, 2013; *Tedesco et al.*, 2013]. Rapid lake drainage events have been argued to either cause substantial channelization of subglacial drainage systems [*Sole et al.*, 2011; *Cowton et al.*, 2013] or interact with preexisting channels [*Howat et al.*, 2010; *Hoffman et al.*, 2011; *Pimentel and Flowers*, 2011]. It is suggested that these channels efficiently remove the water from the injection point and thus limit the dynamic impact of the lake drainage event.

Modeling efforts of Greenland and alpine glacial systems suggest that subglacial channels readily form in regions with steep ice surface slopes, sustained water inputs and thin ice [e.g., *Schoof*, 2010; *Hewitt*, 2011, 2013; *Werder et al.*, 2013]. The bedrock topography in Greenland is sufficiently variable that regional patterns in ice thickness are difficult to constrain [*Griggs et al.*, 2012; *Lindbäck et al.*, 2014]. However, further inland, the surface slopes become more shallow and water inputs become smaller and more widely distributed [*Doyle et al.*, 2014; *Leeson et al.*, 2015; *Poinar et al.*, 2015]. Therefore, following *Bartholomew et al.* [2011b], we partition the Greenlandic drainage systems into an upper (>1000 m above sea level (asl)) and lower (<1000 m asl) ablation area, which we will here refer to as interior and marginal regions, respectively. Most evidence of efficient channelization of basal drainage systems is from the marginal region (e.g., see GPS and tracing data from the lower three to four sites of *Bartholomew et al.* [2010, 2011b], *Cowton et al.* [2013], and *Chandler et al.* [2013]). In contrast, in the interior region, *Bartholomew et al.* [2011b] and *Moon et al.* [2014], for example, found limited evidence of channelization during the melt season with ice velocities varying in phase with changes in water input rates. Similarly, *Chandler et al.* [2013] found distributed systems at tracing sites 41 and 57 km inland on Leverett Glacier (both located in the interior region) with channels only developing at the 41 km site by early August. This corresponds with outputs from simple subglacial channel models by *Dow et al.* [2014a] and *Meierbachtol et al.* [2013] that suggest that channel growth is limited in interior regions, where the hydraulic gradients are weak.

We are interested in whether rapid supraglacial lake drainage events can cause substantial channel formation and whether this, or the subsequent hydrological system development once a surface-to-bed connection has been made, is sufficient to raise the system effective pressure (the ice pressure minus the water pressure). Higher effective pressure suggests less basal lubrication and therefore, in a general sense, lower ice velocities. In particular, we focus on a lake drainage event in the interior of the ice sheet (70 km from the margin) and apply a coupled numerical model of subglacial hydrological development that allows both radial flux expansion in the vicinity of the water input point, and the development of an integrated distributed and channelized drainage system downstream. We combine the results of our modeling with field data collected from the rapidly draining lake site to interpret the subglacial hydrological development, both during and following rapid lake drainage in this region.

## 2. Field Site

**Figure 1:**
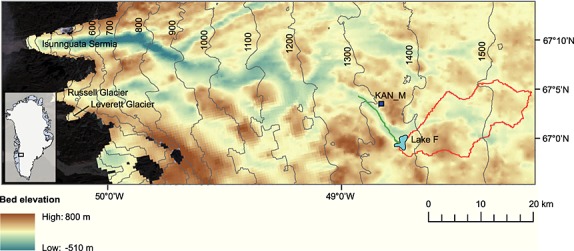
Bed elevation map of the Russell Glacier catchment with surface elevation plotted as 100 m contours. The basal digital elevation model (DEM) was produced following *Lindbäck et al.* [2014]. The background image is a LANDSAT TM output from 18 August 2010. Also plotted are the location of Lake F (light blue), the Lake F catchment area (red), the model flowline (green), and the KAN_M automatic weather station (dark blue).

Our lake drainage field site, hereafter referred to as Lake F, is located at 67.01°N 48.74°W, in the Russell Glacier catchment in West Greenland, ∼70 km from the glacier terminus (Figure 1). Lake F lies at an elevation of 1350 m asl where ice thickness is ∼1200 m; this lake is therefore ideally situated for testing the impact of water access to the bed of thick, interior ice.

**Figure 2:**
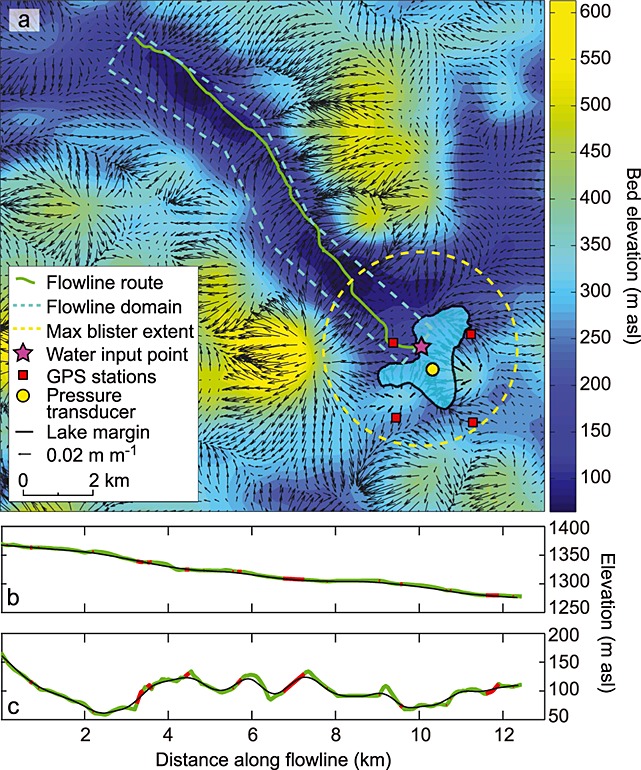
(a) Basal DEM of the Lake F region with hydraulic potential vectors. Hydraulic potential gradients are calculated assuming the lake is full and basal water pressures are everywhere at overburden. The extent of Lake F just prior to drainage is shown in opaque blue. The extent and route of the flowline and blister domains along with the location of the GPS stations, pressure transducer, and the water input point at the bed estimated from passive seismic records are indicated. (b and c) Surface and bed topography along the flowline (green) in Figure 2a with red areas showing regions where hydraulic potential gradients are reversed at overburden pressure. The black curves show the smoothed surface and bed used in the model runs.

The lake site was instrumented and monitored during July and August 2010. Positions of GPS receivers around the lake and the location of the pressure transducer measuring lake water level are shown in Figure 2a. Information on the dynamic response of the ice during lake drainage from records of GPS uplift and vertical motion can be found in *Doyle et al.* [2013]. We use these GPS and lake water level records as constraints on our model outputs.

Immediately prior to drainage, the lake volume was 7.4 × 10^6^m^3^ with a surface area of 2.6 × 10^6^m^2^. The lake began to drain at 01:40 UTC on 30 June (day of year 181) with a maximum drainage rate of 3300m^3^s^−1^ at 02:47 UTC, as determined from pressure transducer water head measurements and a lake bathymetry map. The lake fully drained in 2h10min [see *Doyle et al.*, 2013, Figure 8]. Analysis of hydrofracture using passive seismic techniques (as detailed in *Jones et al.* [2013]) allows triangulation of the water input point at the ice-bed interface. This point is indicated by the pink star in Figure 2a and marks the water input point for our modeled domain.

Subglacial and surface digital elevation models (DEMs) of the region around Lake F have been produced at a resolution of 250 m and linearly interpolated onto a 50 m grid (see *Lindbäck et al.* [2014] for methods). A map of the regional bed topography is shown in Figure 2a. A subglacial valley runs into the region of Lake F from the NE and turns at the lake site, continuing toward the NW; this valley coincides with a fast flow unit indicated in a velocity map of the region produced by *Palmer et al.* [2011]. We have applied hydraulic potential calculations for water pressures at overburden, following the method of *Shreve* [1972], to the surface and bed DEMs with the resulting potential vectors indicated by the black arrows in Figure 2a. The green flowline from the Lake F site in this figure (and Figure 1) plots the water flow route to the NW implied by the hydraulic potential gradients.

## 3. Numerical Models

Our approach couples two models that have previously been applied to a similar lake drainage event that was reported by *Das et al.* [2008]. These models are the following: (1) a turbulent, radial flux and uplift model by *Tsai and Rice* [2010], hereafter referred to as the water blister model, and (2) a 1-D distributed and channelized flowline drainage model that constitutes part of an ice dynamics model by *Pimentel and Flowers* [2011], hereafter referred to as the hydraulic flowline model. Our aim is to address the limitations of each model by coupling them and produce a method to assess hydrological development during lake drainage and throughout the remaining melt season. Here we describe each model in turn, followed by a description of our coupling process.

### 3.1. Water Blister Model

The water blister model [*Tsai and Rice*, 2010, 2012] represents a turbulent sheet of water that flows between the ice and the substrate in a “crack” along a flat bed. In glaciological terms this crack represents hydraulic jacking of the ice and the consequent flux of water through the gap at the ice-bed interface. The extent of hydraulic jacking is established though elastic ice mechanics: in this case, the balance between subglacial water pressure force and resistive elastic forces in the overlying ice. Elastic mechanics are also applied to determine the flux through the englacial fracture that is formed during initial lake drainage. The blister model does not calculate a vertical hydrofracturing criteria (such as that discussed by *van der Veen* [2007]) that would allow initial flux of lake water into the ice subsurface but instead assumes that a surface-to-bed englacial crack already exists and that water has reached the bed prior to model initiation. However, by including a preexisting englacial fracture in the blister model, the modulation of water flow due to frictional resistance of the vertical crack walls is taken into account.

We apply *Tsai and Rice's* [2010] model of a radially expanding water blister that uses a clamped elastic plate in order to determine the expansion of the water blister past radii equal to the ice thickness. For a full treatment of the model equation development, see *Tsai and Rice* [2010]. The primary output of the blister model is the rate of expansion of lake water at the bed, represented by *L*, the blister radius, that increases over time, *t*:


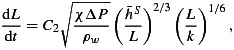
(1)

where *C*_2_ is a constant determined from self-similar analysis [see *Tsai and Rice*, 2010], *χ* is a friction parameter, Δ*P* is the water pressure in the connecting vertical crack, *ρ*_*w*_ is the density of water, 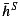 is the average basal opening when applying an elastic plate with clamped edges and uniform loading, and *k* is the Nikuradse channel roughness height. The remaining primary equations and model constants are presented in the supporting information.

The *Tsai and Rice* [2010] model is a novel method for analyzing rapid drainage events, but it is not without limitations. For the purpose of establishing subglacial hydrological characteristics and resulting ice dynamics during and after lake drainage, the water blister model can only contribute to establishing conditions within a short (several hour long) time period. If allowed to run for longer time periods, the modeled radius of the water blister continues to expand considerably further than would be expected in any subglacial system due to topographical variations. Such variable topography is present around the case study lake (see Figure 2). Another limitation is that the water pressure across the blister during growth is assumed to be static, although pressures in the far field might be lower [see *Adhikari and Tsai*, 2015]. The water blister model also cannot account for either a preexisting drainage system or the downstream flow of water from the blister that might contribute to the development of subglacial hydrological systems.

### 3.2. Hydraulic Flowline Model

The hydrological component of the *Pimentel and Flowers* [2011] ice dynamics model incorporates both distributed and efficient water flow, following the approach of *Flowers* [2008]. The distributed system consists of a water sheet with thickness averaged across the fixed width of the hydraulic flowline, and a fixed hydraulic conductivity; as such, it represents a simplified version of a linked-cavity system or a sediment-based drainage system. Vertically integrated water flux in the distributed sheet, *q*^sh^, is calculated with the Darcian flow equation


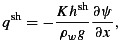
(2)

where *K* is the hydraulic conductivity, *h*^sh^ is the thickness of the water sheet, *g* is the gravitational acceleration, and *∂ψ*/*∂x* is the gradient of hydraulic potential.

The efficient system is based on channel equations for a semicircular channel overlying a hard bed, which grows by viscous dissipation of heat that melts the surrounding ice, offset by inward creep driven by the pressure of the overlying ice. Discharge through a semicircular R-channel, *Q*^*c*^, is described by


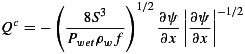
(3)

where *S* is the cross-sectional area of the channel, *P*_wet_ = (*π* + 2)*R* is the wetted perimeter with R the channel radius, 
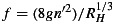
 is the Darcy-Weisbach friction factor with 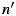 representing the Manning roughness parameter, and *R*_*H*_ = *πR*/(2*π* + 4) the hydraulic radius for a semicircle. The development of the channel cross-sectional area, *S*, is governed by


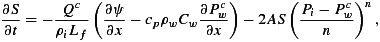
(4)

where *ρ*_*i*_ is the density of ice, *L*_*f*_ is the latent heat of fusion, *ρ*_*i*_ is the ice overburden pressure *A* and *n* are parameters in Glen's flow law, *c*_*p*_ is the specific heat capacity of water, *C*_*w*_ is the Clausius-Clapeyron gradient, and 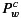 is the water pressure in the channel. The latter three terms allow for pressure-melt dependence in the channel. The hydraulic flowline model has a fixed finite-difference domain geometry with longitudinal temporal evolution and static lateral conditions. We detail the remaining primary hydraulic flowline equations and model constants in the supporting information.

The hydraulic flowline model involves several assumptions that are important to note when applying the model to a rapid lake drainage event. No lateral or radial flow is possible outside the fixed width of the hydraulic flowline; therefore, the flux width has to be estimated. Also, water is input directly from the ice surface to the bed; there is no modulation from englacial flow, either frictionally or elastically. The lack of englacial flux modulation and lateral flow is particularly problematic when introducing the high discharge rates from lake drainage events into the model.

### 3.3. Model Coupling

Our approach to modeling rapid lake drainage is to overcome some of the limitations of the two individual models by applying them in conjunction. Our coupled model simulates the growth of a radial water blister caused by lake drainage that simultaneously drives downstream hydrological development in the flowline model. As a result, (a) the lake water is modulated by englacial flow through the vertical fracture and (b) radial flux during drainage is accounted for. Once all the water has drained from the lake, the characteristics of the blister are used as initial conditions in the hydraulic flowline so that longer term fluxes due to the overpressurized (i.e., above the ice overburden pressure) lake water can be determined. Each lake drainage simulation comprises two stages in sequence as detailed below and in section 4.

#### 3.3.1. Stage I: Lake Drainage

**Figure 3:**
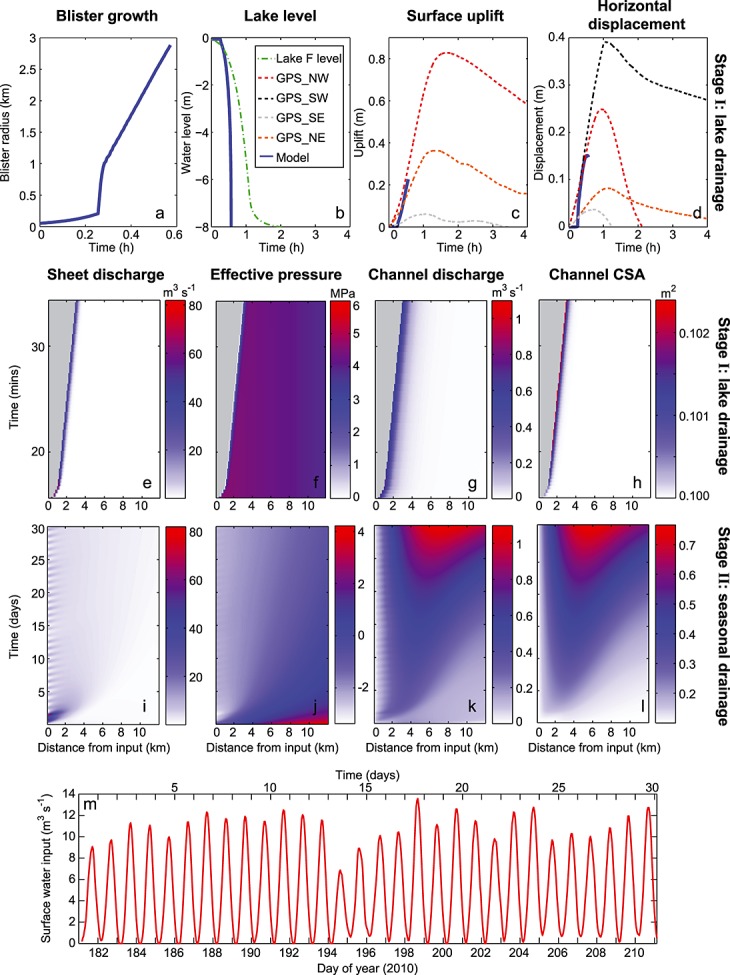
Coupled hydrological model outputs from the baseline *planar geometry* test with an outlet pressure of 0.7 *P*_*i*_. Primary blister model outputs including (a) blister growth, (b) lake level, (c) ice surface uplift, and (d) horizontal (crack opening) displacement, compared with surface GPS data and lake water level data. Note that GPS_SW had little uplift during this period and is therefore not plotted. Flowband distance-time plots showing distributed sheet discharge, sheet effective pressure, channel discharge, and channel cross-sectional area (CSA) for (e–h) the Stage I linked flowline where blister overlap is shown by the gray shading and (i–l) the Stage II hydraulic flowline showing seasonal outputs for the hydraulic flowline. (m) Diurnal surface water inputs used as source terms for Stage II.

The initial period of lake drainage involves large volumes of water reaching the ice-bed interface. The blister model incorporates the mechanical adjustments of the ice as a result of these water pressures above overburden, as suggested by a radial pattern of ice uplift observed at the case study lake [*Doyle et al.*, 2013]. Concurrent downstream flux is calculated using the hydraulic flowline model, with the upstream domain boundary initially located where the vertical fracture from the lake reaches the bed. To best represent these processes, the water blister model is run and linked to the hydraulic flowline model so that (a) the blister incorporates water from the hydraulic flowline domain that it overlaps and (b) water at the blister boundary is removed as a result of flux into the hydraulic flowline (see Figures 3a–3d and 3e–3h). This linkage means that for every blister time step, the hydraulic flowline evolution is also calculated and a flux relationship between the two is determined.

As the blister expands rapidly and creates a strong radial hydraulic potential gradient, we suggest that the water will overwhelm any preexisting channels. One of the limitations of the blister model is that it cannot grow channels within the radius of the blister. It is possible that with the presence of large channels, water could be directed out of the water blister in a preferred direction dictated by the lower hydraulic potential within the channels. However, as shown in *Dow et al.* [2014a], the duration of a rapid lake drainage event (and in this case, blister expansion for less than an hour) is not sufficient to grow large channels. For example, if we apply a constant flux rate of 1.7m s^−1^ (the maximum blister expansion rate from our model runs) to equation (4), we find a channel cross-sectional area growth rate of less than 0.02 m^2^ within the period of Stage I. The lack of channels within the blister will therefore have little impact on our model outputs.

Another limitation of the water blister model is that, for our configuration, it will continue to expand up to a radius of 15–20 km before approaching equilibrium. As the subglacial topography around Lake F does not remain flat over such a distance (see Figure 2), we stop the blister model at the point when all lake water is either within the englacial fracture or in the subglacial system (in the blister or the hydraulic flowline domain). The maximum domain extents of both the blister and the hydraulic flowline are plotted in Figure 2a. At the point when all water has drained from the lake into the subsurface, two parameters are calculated from the blister model and used as inputs in the Stage II model. These parameters are (1) the thickness of the blister calculated at fixed intervals between the water input point and the margin of the blister and (2) the water pressure at the input point, determined by the water column in the englacial system (i.e., the full thickness of the ice).

#### 3.3.2. Stage II: Seasonal Hydrological Development

**Figure 4:**
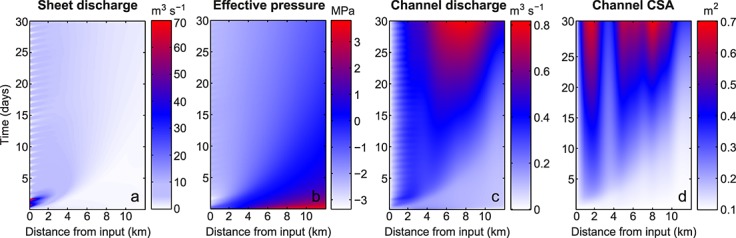
Coupled hydrological baseline model outputs from the *realistic geometry* test with an outlet pressure of 0.7*P*_*i*_. Stage II hydraulic flowline distance-time plots of (a) sheet discharge, (b) sheet effective pressure, (c) channel discharge, and (d) channel cross-sectional area (CSA).

Once all the lake water is in the subsurface, water pressures near overburden will drive flux downstream. For the remainder of the model run, we therefore use the flowline model to assess hydrological development. In the area of the hydraulic flowline domain where the blister has overlapped, the pressure and water thickness is determined by the blister water reservoir. As the flowline width will limit incorporation of only around a third of the blister water at the end of Stage I, we conserve mass by maintaining the volume in the overlapped portion of the flowline until all of the blister water has entered the flowline. In addition, we include surface water inputs into the flowline representing flux into the moulin formed following the lake drainage event. This stage of the model is run for a further 30 days following lake drainage to estimate seasonal development of the hydrological system. See Figures 3i–3l and 4 for examples of the Stage II model outputs.

## 4. Numerical Approach

In this section we describe in detail the methods used to couple the water blister and hydraulic flowline model (Stages I and II).

### 4.1. Stage I: Lake Drainage

In each linked model run, the water blister is allowed to expand to a radius equal to the size of one flowline grid cell (100 m) prior to linkage, for model stability. Once this threshold is reached, the blister model is linked to the hydraulic flowline model. The initial condition of the Stage I hydraulic flowline model is a steady state obtained from an uncoupled model run.

The hydraulic flowline domain initially begins at the location where the vertical fracture from the lake reaches the bed (indicated by the pink star in Figure 1) and as the blister model grows, it “overlaps” the hydraulic flowline model domain. In order to conserve mass, the water in the overlapped flowline domain is incorporated into the blister and therefore removed from the hydraulic flowline. The length of overlap between the two models is established by rounding the blister radius value to the nearest grid cell. This has been tested for grid resolution dependence, and it was found that smaller grid cells made little difference to the model outputs. The volume of water in those overlapped grid cells, *V*_*f*_, is relative to the size of the blister radius, *L*, until the latter equals or exceeds the size of the flowline fixed width, *W*, so that


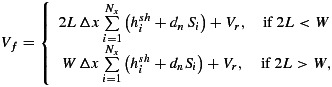
(5)

where Δ*x* is the size of one grid cell, *d*_*n*_ is the number of channels in the hydraulic flowline model (in our case, one), *N*_*x*_ is the number of grid cells overlapped by the blister, and *V*_*r*_ is any volume overlapped as a result of further blister growth over grid cells initially overlapped in previous time steps. *V*_*r*_ is defined as


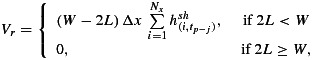
(6)

where *t*_*p* − *j*_ is the primary time step when grid cell *i* was originally overlapped. Because the blister is radial whereas the hydraulic flowline has a rectangular geometry, the calculated overlap volume will not be exact but is a necessary simplification for our configuration.

The second impact of the overlap of the blister into the hydraulic flowline domain is that for each primary time step, the overlapped grid cells are removed from the hydraulic flowline domain. As a result, as the blister grows, the hydraulic flowline domain shrinks. This is a reasonable approximation as it is likely that the growth of the blister will overwhelm any preexisting drainage system. The flux removed from the blister by the hydraulic flowline model is not due to forcing water into the domain as source terms but instead is a result of downstream hydrological development, *V*_out_:


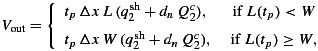
(7)

where *t*_*p*_ is the primary time step, 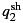 is the sheet flux out of grid cell 2, and 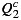 is the channel flux out of grid cell 2. Within one primary time step, the volume change within the blister is therefore *V*_new_=*V*_*f*_−*V*_out_. Because the blister model is based on elastic mechanics, the volume and length of a blister will always be the same for a certain rate of ice uplift in one region (given the same initial conditions). As a result, by knowing the adjusted volume of the blister, the corresponding radius can be calculated within the same primary time step. This is still an approximation as water flux is not included directly within the blister model but is sufficient for our analysis. The upstream boundary pressure condition for the Stage I hydraulic flowline model in both the sheet and the channel, where they intersect with the edge of the water blister, is defined as the ice overburden pressure; this is to overcome the pressure singularity at the blister boundary as discussed by *Adhikari and Tsai* [2015].

### 4.2. Stage II: Seasonal Hydrological Development

The second stage of modeling uses the calculated blister as a water reservoir within the hydraulic flowline model; the blister water is therefore added back into the hydraulic flowline. As a result, the overpressure that still remains due to water in the connecting englacial crack is used to drive hydraulic flowline development.

The initial condition for hydraulic flowline sheet water thickness (*h*^sh^) is calculated from (a) the blister water thickness at each grid cell over a distance equivalent to that overlapped in Stage I and (b) the water height from the remainder of the domain in the Stage I hydraulic flowline. The blister thickness approaches zero toward the radial margin; however, it is assumed that the edge of the blister represents overburden pressure due to the presence of a preexisting drainage system. As a result, the blister water thickness is adjusted so it approaches the critical water thickness (where pressure in the sheet is at overburden) at the radius margin as opposed to zero water thickness. The Stage II initial channel size within the blister reservoir is assumed to be the same cross-sectional area as the channel at the blister radial margin (i.e., in the first grid cell at the final time step of the Stage I flowline); the initial condition channel size in the downstream remainder of the flowline is determined from the Stage I hydraulic flowline output.

During the seasonal development, surface water is input into the flowline as a boundary source term. As the moulin will likely connect directly with the flowline channel, the surface input water is added as a channel source term when pressure in the channel is less or equal to overburden. When pressure is greater than 110% of overburden in the channel, uplift would allow water to flow out laterally into the sheet until the channel pressure dropped toward overburden. As a result, if the channel pressure exceeds 110% of overburden, surface water is instead added as a source term to the sheet. Between overburden and 110% of overburden, water is partitioned linearly between the sheet and the channel so that with pressure closer to overburden, more of the water input is received by the channel and vice versa. We have chosen the channel input cutoff as 110% of overburden because, above this level, water would no longer flow into the moulin and would instead pool on the surface. The latter scenario does occur at our lake site for a short period following lake drainage, but the reformed lake has drained again within 12 h.

We estimate seasonal diurnal water input from a surface energy balance model run with weather station observations [*van As et al.*, 2012] and applied to the Lake F catchment (shown in Figure 1). Our estimates of surface melt are calculated using weather station data from a site 10 km to the NW of Lake F (site KAN_M, see Figure 1). The surface melt was calculated using measurements of net shortwave radiation, net longwave radiation, sensible heat flux, latent heat flux, and subsurface conductive heat flux [see *van As et al.*, 2012]. This rate was then extrapolated in bulk to the entire Lake F catchment. We apply a smoothing to prevent fluxes dropping to zero during the night to account for delayed flux over the surface. This is a rough approximation of water fluxes into the system as it does not account for surface storage and transport [e.g., *Banwell et al.*, 2012; *Clason et al.*, 2015]. However, it is sufficient for estimating a diurnal water flux into our modeled system. The water input is plotted in Figure 3m in relation both to the day of year and the time within the model runs. We also run a sensitivity test by halving the input rate to the system.

### 4.3. Model Limitations

Our modeling approach has some limitations. The method of overlapping the radial blister model with the rectangular hydraulic flowline model in Stage I is not exact. Similar dimensional problems exist when running the Stage II hydraulic flowline model with the blister reservoir. For example, blister water that radially expanded upstream and outside of the flowline fixed width is not explicitly forced into the hydraulic flowline model but instead is used to replace water that flows out of the overlapped flowline until the blister reservoir is exhausted. Although this conserves mass, it will not be a fully accurate representation of the fluxes at the bed of the ice following drainage. The lack of nonuniform expansion of the blister also prevents accurate flux direction calculations. Other limitations that are not resolved in our modeling include lack of lateral flux in the flowline model and some inaccuracies in the hydrofracture solution when applying the blister model to radii greater than the ice thickness (as detailed in *Tsai and Rice* [2010]). These are all issues that put caveats on our model outputs and provide incentive for application of a more complex 2-D or 3-D model to the rapid lake drainage scenario. Despite these limitations with our approach, we believe our outputs provide a general understanding of the hydrological conditions and encourage greater investigation, from both data collection and modeling, into these systems.

## 5. Model Configuration

The data collected during the 2010 field campaign at Lake F allow some of the coupled model inputs to be constrained with a reasonable degree of certainty. The width of the flowline for all model runs is fixed at 1500 m as this is the average width of the valley running downstream from the water input point toward the NW (Figure 2a). Some adjustments are made to the *Tsai and Rice* [2010] water blister model when applied to the Lake F drainage event. As we have an accurate lake bathymetry map, it is possible to reasonably constrain the modeled lake water height by adjusting for lake volume change. Other known inputs to the water blister model include the ice thickness at the water input point and the positions of the four GPS units relative to the water input point. The GPS uplift and horizontal motion records from the lake drainage event are compared with outputs from the blister model, which are extracted in relation to the specific locations of the individual GPS. Following *Pimentel and Flowers* [2011], we have one channel in the domain extending from the moulin input. The flowline grid resolution is 100 m, having determined with sensitivity tests that smaller grid cells do not affect the model outputs.

The impacts of the remaining unknown variables on drainage development are explored in a series of sensitivity experiments. These variables include channel roughness, the critical water sheet thickness (i.e., when the sheet reaches overburden pressure), the conductivity of the distributed system, the water exchange rate between the channels and distributed system, and the initial channel size. The coupled hydrological model is run with a set of baseline parameters (Table 1) following the approach and values selected by *Pimentel and Flowers* [2011]. We deviate from the baseline values of *Pimentel and Flowers* [2011] in the choice of initial channel size (our value is 10^−1^ m^2^ rather than the *Pimentel and Flowers* [2011] value of 10^−3^ m^2^) and the sheet-channel exchange parameter (which we increase to 0.8 from the *Pimentel and Flowers* [2011] value of 0.1). Both parameter adjustments are to allow greater channel growth at the bed as a very small initial channel size will inhibit development and a higher exchange parameter will allow greater flux from the sheet into the channel during the blister development. Choice of the exchange parameter is arbitrary, but we test the relationship within the sensitivity tests, and we also run a sensitivity test with a smaller initial channel size. For the sensitivity tests, each variable is adjusted with respect to the baseline parameters to determine the independent effect of that variable on the hydrological development (the parameter variations are shown in Table 1). We also run a test with the parameters most suited for channel growth to get a maximum channel growth rate. Tests of the values for Young's modulus for ice, Nikuradse roughness height, geothermal heat flux, and creep parameter were found to have little impact on hydrological development and are therefore not discussed in section 6.

**Table 1:** Baseline and Sensitivity Model Parameters

		Baseline	Sensitivity	
Symbol	Description	Value	Values	Units
*A*	Creep parameter	1.6	0.35; 0.93; 2.4	Pa^−3^ s^−1^ ×10^−24^
*E*	Young's modulus	6.2	3; 8.84	GPa
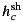	Critical sheet thickness	0.15	0.05; 0.3; 0.4	m
*K*	Sheet hydraulic conductivity	0.1	0.05; 0.5	m s^−1^
*k*	Nikuradse roughness height	0.01	0.05; 0.1	m
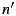	Manning roughness coefficient	0.032	0.01; 0.07; 0.1	m^−1/3^ s
*Q*_*G*_	Geothermal heat flux	70	85	mW m^−2^
*S*_*ɛ*_	Initial channel size	0.1	0.01; 1; 10	m^2^
*X*^*s*:*c*^	Sheet-channel coupling coefficient	0.8	0.1; 0.5; 1	Dimensionless

### 5.1. Model Geometry

We perform model runs with two different geometries: one with simple *planar* topography and the second with more *realistic* geometry for the Lake F region. The purpose of two geometries is to allow an assessment of hydrological development at Lake F (with the realistic geometry) but also allow a wider application of our findings so that the dynamic impact of lake drainage events and access of water to the bed in similar regions can be analyzed.

The planar geometry has constant surface and bed slopes of 0.45° and 0.14°, respectively, that extend for 12 km downstream from the water input point. To avoid boundary effects, the spatial domain is arbitrarily extended by a farther 12 km, but the outputs are only examined up to the edge of the 12 km domain. The ice thickness at the upper boundary is 1230 m and at the lower boundary at 12 km distance is 1170 m, both estimated from the Lake F DEMs. This planar geometry allows analysis of hydrological development without the presence of reverse bedrock slopes, which complicate interpretation. The planar geometry tests are applied with two prescribed outlet pressure values (*P*_out_) at the downstream boundary of the hydraulic flowline: (1) ice overburden (*P*_*i*_) and (2) 70% of overburden. These boundary conditions are based on the range of water pressures found from borehole studies closer to the margin of the ice sheet by *Meierbachtol et al.* [2013] and *Andrews et al.* [2014]. It is likely that, further inland where we run our model, 70% of overburden is too low for an outlet boundary pressure condition, but nevertheless, we include it as a lower bound to produce conditions conducive for hydrological development.

The realistic geometry is based on the surface and basal topographies at Lake F extracted along the flowline shown in Figure 2a and are plotted in Figures 2b and 2c. The reverse bedrock slopes along the flowline are sufficiently steep that they would cause numerical instabilities in the coupled hydrological model by producing reverse potential gradients that would cause water flow upstream. For modeling purposes, the flowline surface and bed topography are smoothed sufficiently to remove the effect of reverse potential gradients (for water pressures at overburden), although the adverse slopes are still, to some extent, preserved (see black curves in Figures 2b and 2c and red curves for regions of reverse potential gradients). The ice thickness at the upper boundary is 1215 m and 1165 m at the lower boundary. Again, the model domain ends at ∼12 km distance but is artificially extended by 12 km to remove boundary pressure effects; the extended portion of the spatial domain follows a constant basal slope of 0.07° and a constant surface slope of 0.22°. The outputs from the realistic geometry are also only analyzed in the initial 12 km. Like the planar geometry model, the realistic geometry model is run with prescribed outlet pressure conditions (*P*_out_) of overburden and 70% of overburden.

## 6. Results and Analysis

**Figure 5:**
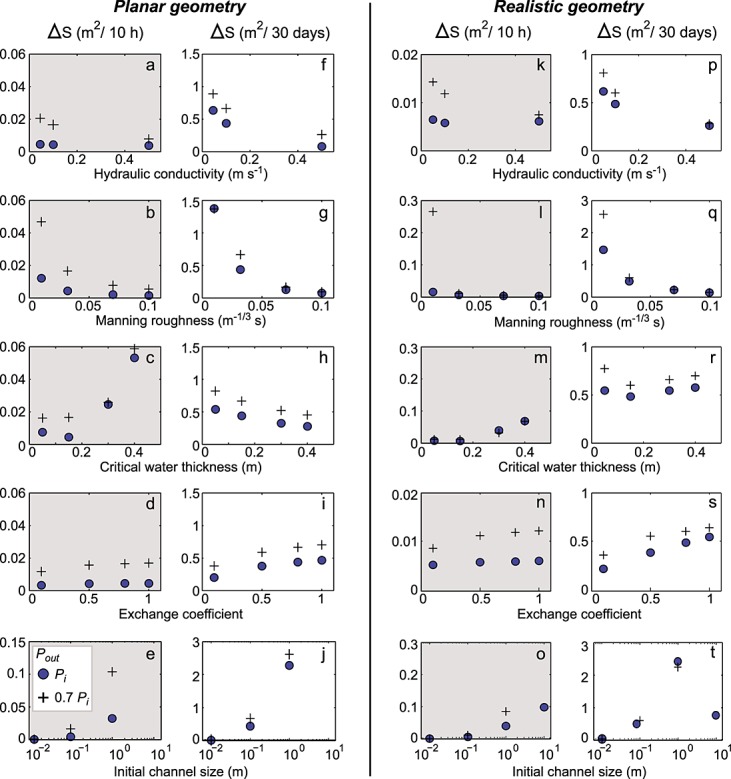
Change in channel cross-sectional area (Δ*S*) over 10 h and 30 days following lake drainage initiation for the planar geometry and the realistic Lake F geometry. Outputs for system outlet water pressures of *P*_*i*_ and 0.7 *P*_*i*_ are plotted as blue circles and black crosses, respectively. Each plot represents channel growth when varying one parameter from baseline values. Note the differences in scale between the axes and that any channel shrinkage is not plotted.

**Figure 6:**
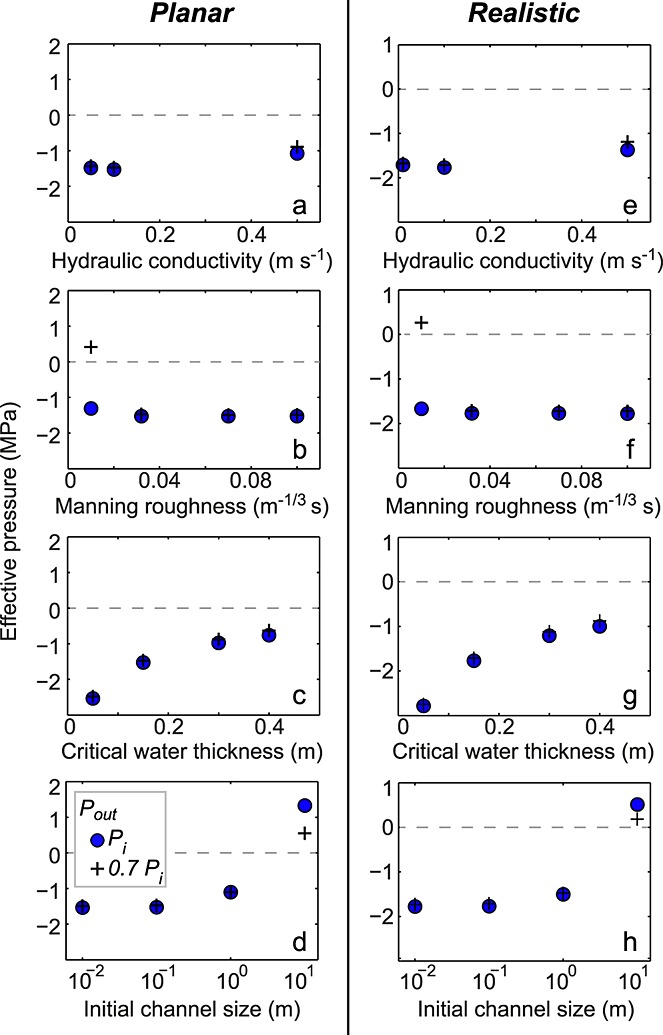
Average effective pressure in the distributed system at 30 days following lake drainage for system outlet water pressures of *P*_*i*_ and 0.7 *P*_*i*_, plotted as blue circles and black crosses, respectively.

We focus on channel development (as measured by temporal change in subglacial channel cross-sectional area) and the changes in the distributed system effective pressure to examine hydrological development in the vicinity of the lake drainage event. By assessing rates of channel growth and the impact of this on effective pressure, we can investigate whether an efficient drainage system is likely to develop in interior regions of the GrIS and thus make a step toward predicting the longer term dynamic impacts of basal water input to these regions. We present the maximum growth in channel size for each sensitivity test for the time periods of (1) 10 h following lake drainage, to allow assessment of drainage development as a direct result of lake drainage, and (2) 30 days following lake drainage, to examine drainage development due to the combination of lake drainage and diurnally variable surface water input to the bed (Figure 5). Results of the mean and maximum channel growth and their related parameters, along with the baseline results, can be found in Table 2. We also plot the average effective pressure in the distributed system, at 30 days following the lake drainage, for the sensitivity tests (Figure 6). Unless otherwise specified, the initial channel cross-sectional area is 0.1 m^2^. Our results can be compared with channel cross-sectional area change modeled for alpine glaciers which have been calculated to grow by ∼0.5–0.75 m^2^ over 30 days from an undeveloped system [*Cutler*, 1998; *Hewitt*, 2011; *Pimentel and Flowers*, 2011]. We first present the baseline parameter results and then discuss the results from the sensitivity tests.

**Table 2:** Channel Growth Rates and Associated Parameters for the Baseline, Sensitivity, and Maximum Channel Tests

Model		Mean Δ*S*	Maximum Δ*S*		Mean Δ*S*	Maximum Δ*S*	
Geometry	*P*_out_	(m^2^/10 h)	(m^2^/10 h)	Parameter	(m^2^/30 d)	(m^2^/30 d)	Parameter
Planar	1	0.00	0.00	Baseline	0.30	0.43	Baseline
	0.7	0.01	0.02	Baseline	0.53	0.67	Baseline
Realistic	1	0.00	0.01	Baseline	0.27	0.48	Baseline
	0.7	0.00	0.01	Baseline	0.42	0.60	Baseline
Planar	1	0.00	0.05	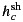 = 0.4 m	1.78	2.28	*S*_*ɛ*_ = 1 m^2^
	0.7	−0.10	0.11	*S*_*ɛ*_ = 1 m^2^	1.80	2.61	*S*_*ɛ*_ = 1 m^2^
Realistic	1	−0.06	0.11	*S*_*ɛ*_ = 10 m^2^	1.24	2.41	*S*_*ɛ*_ = 1 m^2^
	0.7	0.08	0.27	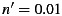 m^−1/3^*s*	1.44	2.57	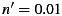 m^−1/3^*s*
Planar	0.7	0.02	0.05	Max test	1.88	2.08	Max test
				*S*_*ɛ*_ = 0.1 m^2^			*S*_*ɛ*_ = 0.1 m^2^
	0.7	0.07	0.1	Max test	0.35	0.51	Max test
				*S*_*ɛ*_ = 1 m^2^			*S*_*ɛ*_ = 1 m^2^

### 6.1. Baseline Results

The baseline model runs produced a maximum blister radius of ∼2880 m at ∼30 min following the period when lake water first reached the ice-bed interface for both model geometries (Figure 3a). This maximum blister radius fits well with the likely extent of lake water flux as suggested by the hydraulic potential gradients (Figure 2a). The modeled drainage rate of the lake is faster than the measured rate (Figure 3b), suggesting that the modeled blister also expands faster than it does in reality. Recent modeling by *Rice et al.* [2015] suggests that creep opening of the englacial hydraulic fracture prior to rapid lake drainage produces a better match between measured and modeled lake drainage rates, but as we assume the lake hydrofracture opens only at the beginning of lake drainage, we do not include this process. The maximum uplift produced with the blister model is less than that seen by the GPS. Uplift for the baseline model runs is ∼0.23 m, whereas GPS_NW, for example, sees uplift of ∼0.8 m (Figure 3c), although some of this motion can be attributed to ice faulting [*Doyle et al.*, 2013]. A similar underestimation of surface uplift was experienced by *Tsai and Rice* [2010] when applying the blister model to the *Das et al.* [2008] lake drainage. GPS_NW uplift was more than twice the uplift seen at the other GPS sites, suggesting that water primarily flowed to the NW along the subglacial flow path implied by the geometric hydraulic potential gradients (Figure 2a). However, the uplift of other GPS units indicates that water did, to some extent, flow radially from the input point. Although the lack of topographical control on the blister expansion model could impact the coupled hydrological model outputs, the shape of the uplift curve matches well with those recorded in the field, as can be seen in Figures 3c and 3d; it is only the magnitude of uplift that cannot be fully replicated. Only partial uplift and horizontal crack opening model outputs are plotted because this is the extent of the blister during the Stage I calculations prior to switching fully to the hydraulic flowline model (Stage II).

Model outputs for seasonal (Stage II) channel development in the planar and realistic geometries are plotted in Figures 3i–3l and 4, respectively. Channel cross-sectional area can been seen to be much more spatially variable for the realistic geometry compared to the planar geometry (Figures 3l and 4d). This is linked closely to the spatial differences in topography for the realistic geometry, as shown in Figures 2b and 2c. During the lake drainage and as a result of blister expansion, the distributed system becomes pressurized resulting in negative effective pressures in both geometries (Figures 3j and 4b).

In both geometries, there was ≤0.01 m^2^ of channel growth over 10 h for all pressure tests. Over 30 days, with *P*_out_ = 0.7*P*_*i*_ the channel could grow by 0.67 m^2^ (Figure 3l). When *P*_out_ =*P*_*i*_, channel growth over 30 days was ∼0.48 m^2^ for both geometries. Therefore, for the baseline parameter runs, which are our best guess scenario for basal conditions at the case study site, there is little channel growth during lake drainage (Figure 3h). Some channel growth occurred over the 30 days following the lake drainage event, but not enough to raise the distributed system effective pressure (see Figure 6). Instead of channels, it is the distributed drainage system that dominates the basal hydrology. During and following the lake drainage event, sheet discharge across the flowline width is more than an order of magnitude greater than that in the channel (compare Figures 3e and 3i with Figures 3g and 3k and Figures 4a and 4c).

The very low effective pressures seen in the baseline and sensitivity tests (Figure 6) suggest that the volume of surface water input into the system is too high for the fluxes possible in the hydrological network. In reality, such high water pressure would cause ice-bed separation and spreading of a water sheet until the pressures are reduced [*Iken et al.*, 1983]. This is not a process that is currently readily applicable in flowline hydrology models. It is also possible that more accurate calculations of the rates of seasonal surface water input into the system incorporating surface flux delays (and therefore reducing water input rates at periods of high melt) would allow higher effective pressures. However, low effective pressures in the distributed system encourage channel growth and are therefore unlikely to hinder system development.

### 6.2. Planar Geometry Sensitivity

As the basal conditions are largely unknown, we present results from a range of sensitivity tests to assess the importance of different parameters on channel growth (Figure 5). Over 10 h of drainage, channel growth is greater for the system with an outlet pressure of 0.7*P*_*i*_ and is most significant in the system with smooth channel walls or a thicker critical water depth (i.e., a Manning roughness value of 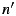 = 0.01m^−1/3^ s or a critical water thickness of 0.4 m; see Figures 5b and 5c). However, over 10 h there is <0.06 m^2^ of channel growth for all of the planar geometry tests, regardless of outlet pressure (see Figures 5a–5d). The exception to this is the test where the initial channel size is 1 m^2^, when maximum channel growth is 0.11m^2^ (although mean channel growth is −0.10 m^2^; see Table 2). Over this time period, the distributed drainage system therefore dominates the removal of lake drainage water in this model geometry.

Over 30 days of drainage, with diurnal water inputs, greater channel growth occurs (Figures 5f–5j). If no preexisting drainage system is presumed prior to lake drainage (represented here with an initial channel size of 0.1m^2^), smooth channels allow the greatest growth of 1.37 m^2^ over 30 days for both outlet pressures (Figure 5g). If a preexisting channel of 1m^2^ is assumed prior to lake drainage, channels can grow by 2.61m^2^ over 30 days when outlet pressures are at 0.7*P*_*i*_ (Figure 5j). If the preexisting channel size is assumed to have a large cross-sectional area of 10 m^2^, these channels shrink throughout the model runs as there is not enough water to keep the channels pressurized.

For the planar model runs, the effective pressure is consistently low for most tested variables apart from a very smooth channel or for systems with preexisting channels of 10 m^2^, where positive effective pressures occur after 30 days of drainage (Figures 6a–6d).

Tests on the lake volume with the baseline parameters and an outlet pressure of 0.7*P*_*i*_ show that an error of ±10^6^ m^3^ produces a change in blister radius of ±200 m, an error for channel growth after 10 h of ±1 cm^2^ and after 30 days of ±0.18 m^2^. We also ran the baseline tests with half the surface runoff and found this reduced the growth of channels by 0.15 m^2^ and 0.85 m^2^ for the systems with initial channel sizes of 0.1 m^2^ and 1 m^2^, respectively.

### 6.3. Realistic Geometry Sensitivity

The realistic geometry is based on the surface and basal DEMs for the Lake F region and produces outputs specific to this region. Mean channel growth is generally less in the realistic geometry compared to the planar geometry, due to the presence of reverse slopes in the former limiting channel growth (see Table 2).

Assuming no preexisting drainage system, channel growth over 10 h is <0.07 m^2^ for all sensitivity tests, unless the channel is very smooth and the outlet pressure is 0.7*P*_*i*_, in which case greater channel growth of 0.27 m^2^ occurs (see Figures 5k–5o).

Over 30 days with no preexisting drainage system, channel growth is <0.80 m^2^ unless a very smooth channel is present, which allows substantially greater growth of ∼2.57 m^2^ when the outlet pressure is 0.7*P*_*i*_ (Figure 5q). Lower outlet pressures again generally allow more channel development for the realistic geometry, due to the stronger pressure gradient that this imposes. However, with an initial channel size of 1m^2^, the system with the higher pressure outlet produces greater channel growth of 2.41 m^2^ over 30 days when compared to the lower pressure system (Figure 5t). With an initial channel size of 10 m^2^ and an outlet at overburden, channel growth occurs; this is in contrast to the other systems where the channel shrinks (Figure 5t). The reverse slopes in this case act to allow channel growth by preventing removal of water from the system and allowing higher water pressures to be maintained, as can be seen from the lower average effective pressures in the realistic geometry when compared to the planar geometry outputs (Figure 6). It is only the systems with an initial channel size of 10 m^2^ or a smooth channel that can raise the system effective pressure above zero after 30 days (Figures 6e–6h).

### 6.4. Maximum Channel Test

We run the planar geometry model with the parameters that are most likely to cause channel growth according to our sensitivity tests. These are a low-sheet hydraulic conductivity (0.05 m s^−1^), a smooth channel (manning roughness value of 0.01m^−1/3^ s), an outlet pressure of 0.7*P*_*i*_, and an exchange coefficient of 1. The critical water sheet thickness was maintained at 0.15 m because of the varied temporal response seen from the sensitivity tests. After 30 days the channel growth is 0.78, 2.08, and 0.51m^2^ for channels of initial size 0.01, 0.1, and 1m^2^, respectively. This can be compared with the growth for the baseline runs of 0.02, 0.67, and 1.64 m^2^. It is interesting that the growth of the 1m^2^ channel was less for the maximum growth parameters. This is because the maximum growth channel became efficient and removed much of the water from the system around 15 days earlier than the baseline channel, thus increasing the system effective pressure enough that the channel began to close. The average effective pressure in the sheet at 30 days is still negative for the very smallest channel but is positive for the two larger initial channels. The choice of parameters is clearly important for determining system development.

### 6.5. Surface Slope Test

**Figure 7:**
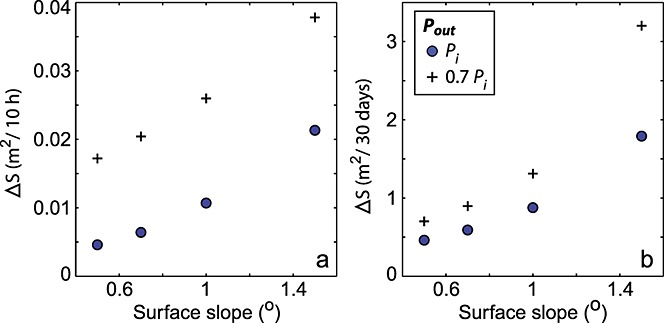
Outputs for the baseline planar geometry model runs with different constant surface slopes: (a) growth in channel cross-sectional area over 10 h following lake drainage initiation and (b) channel growth over 30 days following lake drainage. Outputs for the systems with outlet water pressures of *P*_*i*_ and 0.7*P*_*i*_ are plotted as blue circles and black crosses, respectively.

We test the baseline Lake F planar model with surface gradients ranging between 0.5 and 1.5° to determine the importance of surface slope on channel development. Our upper value is similar to the surface slope near the margin of Russell Glacier (see Figure 1) but is less than those typically found on mountain glaciers. During the 10 h following lake drainage, the channel cross-sectional area expands more quickly for steeper slopes, although in all model runs channel cross-sectional area growth is <0.04 m^2^ over 10 h (Figure 7a). Channel growth over the 30 days following lake drainage is again greatest for steeper surface slopes, particularly for the *P*_out_ = 0.7*P*_*i*_ system where channels can grow by ∼3.20 m^2^ (Figure 7b). Comparing these surface slope results with the sensitivity test results in Figure 5, it can be seen that a steeper surface slope can have as much, if not more, influence on channel growth as the basal system parameters.

## 7. Discussion

By combining the outputs from the modeling sensitivity tests with the data collected in situ at the Lake F drainage site it is possible to hypothesize about the primary characteristics of the rapid drainage event and the impact of lake drainage events on seasonal ice dynamics. In the following section we propose an outline of events during and immediately following drainage, along with a discussion of seasonal evolution of the hydrological system after a surface-to-bed connection has been made. Finally, we discuss the potential implications of our hydrological assessment for ice dynamics.

### 7.1. Lake Drainage

Our model results indicate that large and efficient channels do not form rapidly as a result of lake drainage in regions similar to our case study. Maximum channel growth within 10 h of drainage for the baseline parameters is 0.02 m^2^ (if no preexisting drainage system exists, i.e., an initial channel size of 0.1m^2^); with a smooth channel (i.e., a Manning roughness value of 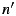 = 0.01m^−1/3^ s) in the realistic system, there is greater but still minimal growth of 0.27 m^2^ over 10 h (Table 2). This suggests that the assertion that large channels form during lake drainage as a result of the large volumes of water reaching the ice-bed interface [*Sole et al.*, 2011; *Cowton et al.*, 2013] is not applicable to lake drainage events in sites similar to Lake F. It is possible that some hydrological development could have occurred prior to the drainage of Lake F. For example, a small (∼1m diameter) moulin to the NW of Lake F was observed to intake some discharge prior to lake drainage (see M4 in Figure 2: *Doyle et al.* [2013]). It is due to this possibility of water access to the bed prior to lake drainage that the results from the 1m^2^ initial channel size tests could be relevant for the Lake F drainage. However, the model results also suggest that very large preexisting channels are unlikely to occur in the Lake F region, as indicated by the mean shrinkage of the 10 m^2^ channels after 30 days of water input in all of the model runs. This shrinkage is due to a lack of available water to maintain pressure in larger channels. The uplift of the GPS units, particularly in the NW (Figure 3c), also indicates that the basal drainage system was not sufficiently hydraulically conductive to remove the water and thus implies that substantial preexisting channels were not present at the time of lake drainage.

Instead, we suggest that at the initial stages of lake drainage, water intersected a distributed system at the ice-bed interface, and, due to the overpressure and limited basal hydraulic conductivity, the ice was hydraulically jacked from the bed. This allowed the spread of a water blister radially from the input point. Due to the basal topography, the blister could only extend radially from the lake up to a radius of ∼2500 m (Figure 2a); beyond this, steep basal slopes prevented further flow. As a result, water was then primarily directed downstream through the subglacial trough to the NW (Figure 2a). However, as the coupled hydrological model outputs suggest, during the period of lake overpressure and drainage, there would likely be little channel formation; the water would instead be drained by a turbulent sheet. Beyond the boundary of the high-pressure turbulent sheet, the hydraulic flowline model outputs suggest that the distributed system was capable of removing the water from the region in the absence of efficient channels (Figures 3e and 3g). Such flux through the blister and the distributed system at the time of lake drainage maintains low effective pressures in the majority of our model runs. Existence of distributed drainage systems capable of removing water fluxes from moulins prior to growth of efficient channels have also been successfully modeled by *Hoffman and Price* [2014] for alpine glaciers.

There is evidence from other studies that water from rapid lake drainage events is evacuated by turbulent water flow and flux through distributed hydrological systems. *Bartholomew et al.* [2011a] measured several spikes in electrical conductivity concurrent with rises in discharge from the Leverett Glacier outlet river in 2009. They attributed these data to water originating from rapidly draining supraglacial lakes flowing through an inefficient drainage system and mobilizing solute-rich water, prior to exiting the glacier. The rapid lake drainage events in Greenland resemble some jökulhlaups where the water input is too large to be contained by channels and instead flows rapidly at the ice-bed interface as a sheet, causing uplift and transient pressure waves [e.g., *Tweed and Russell*, 1999; *Johannesson*, 2002; *Flowers et al.*, 2004; *Sugiyama et al.*, 2007]. These jökulhlaups are not associated with large channel growth [*Johannesson*, 2002; *Flowers et al.*, 2004; *Sugiyama et al.*, 2007]. For example, the Grímsvötn jökulhlaup of 1996 involved peak discharge of flood waters at the outlet within 14 h of a subglacial eruption as a result of turbulent sheet flow [*Roberts et al.*, 2000]. The drainage of ice marginal Gornersee on Gornergletscher, Switzerland, has also demonstrated characteristics of subglacial turbulent sheet flux and hydraulic jacking, similar to the Lake F drainage [*Sugiyama et al.*, 2008]. This turbulent sheet drainage contrasts with the type of jökulhlaup that involves slower leakage of water over the period of days to weeks, which allows large channels to grow and produces exponentially rising discharge in the proglacial outlet hydrographs [e.g., *Nye*, 1976; *Clarke*, 1982; *Sturm and Benson*, 1985; *Walder and Costa*, 1996; *Johannesson*, 2002; *Ng and Björnsson*, 2003]. Very large channels are possible in the latter configuration due to the constant and plentiful supply of water over a much longer period of time than associated with rapid lake drainage events in Greenland [*Clarke*, 2003; *Roberts*, 2005].

### 7.2. Seasonal Drainage

The Lake F rapid drainage event created a surface-to-bed pathway that continued to deliver water inputs to the bed for the remainder of the melt season. According to our model outputs, such sustained and diurnally varying water inputs to the basal hydrological system allows some channel growth in the Lake F region, although the rate is highly dependent on the choice of basal parameters, which are difficult to precisely constrain (Figures 5f–5j and 5p–5t). The maximum channel growth from the Lake F baseline tests (with an initial channel size of 0.1m^2^) is 0.67 m^2^ over 30 days. This growth increases to 2.61 m^2^ if the initial channel size is 1m^2^ for the Lake F planar geometry. The other parameter that causes significant channel growth is the Manning roughness, with a very smooth channel allowing growth of 2.57 m^2^ in the realistic geometry (see Table 2).

As mentioned above, typical valley glacier channel growth rates over 30 days from an undeveloped system have been modeled to be ∼0.5–0.75 m^2^ [*Cutler*, 1998; *Hewitt*, 2011; *Pimentel and Flowers*, 2011]. Our model finds comparable results for the GrIS interior and greater growth when a preexisting channel is present or when the channels are very smooth. The alpine glacier channels could grow with much less water input than we have in our system (in some cases, more than an order of magnitude less, e.g., *Cutler*, [1998]), suggesting the geometry of our case study site (with shallower surface slopes and thicker ice) limits greater channel growth. Our outputs also suggest the presence of a preexisting channel of 1m^2^ at the time of the lake drainage would likely have required sustained water input for more than 30 days from upstream regions. Given that lake drainage events and moulin formation generally occur in bands of increasing elevation [*Liang et al.*, 2012; *Fitzpatrick et al.*, 2014], it is unlikely that sufficient basal water flux through the lake drainage site was available to create a 1m^2^ channel. The channel smoothness is therefore the most likely variable that the modeling suggests could cause significant differences in channel growth in this region, and without in situ data we cannot determine what Manning roughness value is the most applicable.

Despite some channel growth, the system for most of our model runs is not sufficiently efficient to increase the effective pressure to positive values in the distributed system. It could be argued that our water influx is too high for the system so that it becomes overpressurized; although greater water input encourages more channel growth than a system with less water input, it also causes low effective pressures. However, our tests with half the volume of surface water input into an undeveloped system still have somewhat low effective pressures (although in the system with a channel of 1m^2^, this increases above zero after 22 days of water input, which can be compared with 27 days for the full water input).

Previous studies of Greenland subglacial drainage systems also support the results of our modeling work. The SF_6_ traces by *Chandler et al.* [2013] indicated the presence of a distributed system downstream of a moulin located 57 km from the outlet of Leverett Glacier, with a maximum water speed of 0.22 m s^−1^. Their tracing experiments at a moulin 41 km from the outlet suggest development of a channel by the beginning of August with maximum water speeds of 1.04 m s^−1^; prior to this all tests yielded water velocities of <0.4 m s^−1^. This can be compared with the average velocities from the channels in our model runs, which after 30 days of drainage were 1.33 m s^−1^ (for an average channel size of 0.63 m^2^) and 1.13 m s^−1^ (for an average channel size of 0.34 m^2^), for outlet pressures of 0.7*P*_*i*_ and *P*_*i*_, respectively. Thus, large channels are not necessary to transport water quickly. Nevertheless, it is the total water volume flux (that *is* dependent on channel size) and the efficiency of those channels in removing water from the surrounding pressurized distributed system that have most impact on ice dynamics [*Andrews et al.*, 2014].

### 7.3. Lack of Efficient Channels

The Lake F realistic geometry results are based on a smoothed topography extracted from surface and basal DEMs of the region. If the topography is not smoothed along the flowline, the water encounters reverse bed slopes that are sufficiently steep to prevent water flow at overburden pressure. In particular, the flowline ends in a large overdeepening that would also hinder channel formation downstream of our domain. Even with the smoothing, the impact of the reverse bed slopes can be seen in the model outputs. Figure 4d shows rapid spatial transitions between actively growing channels and minimum channel size, dependent on the local topography; this is due to low hydraulic potential gradients on the reverse slopes that result in slow water flux and supercooling freeze-on, which narrows the channel (Figure 4c). When water flows from an area of high pressure to low pressure, it adjusts to the rise in pressure melting point by forming frazil ice, i.e., supercooling [e.g., *Alley et al.*, 1998]. This ice accretion on adverse bed slopes can rapidly shrink channels and hamper efficient drainage by reducing hydraulic conductivity on reverse slopes [e.g., *Creyts et al.*, 2013; *Dow et al.*, 2014b], as seen in the Lake F model outputs. The hydrological system would then act more like an inefficient distributed system despite some channel growth between the adverse slopes. This is illustrated by the mean channel growth rates (Table 2) and low effective pressure in the realistic geometry domain (see Figures 6e–6h). The combination of reverse hydraulic potential slopes, supercooling freeze-on, and rapid channel closure likely act together to create a highly transient and often inefficient drainage system. These results indicate that more attention is required to examine flow over variable basal topography if subglacial drainage development is to be well understood.

The Lake F regional hydraulic potential vectors are shown in Figure 2a and demonstrate that little gravitational potential (a gradient of only ∼0.02 m m^−1^) drives water flux under circumstances where water pressures are at overburden. The gentle surface slope therefore appears to be a key control preventing efficient drainage system development, as demonstrated by our slope tests showing that channels can grow significantly faster with steeper surface slopes (Figure 7). This is supported by the modeling work of *Meierbachtol et al.* [2013], who also found channel growth rates in shallow surface slope ice to be minimal. During a lake drainage event, the gravitational hydraulic potential gradient is overwhelmed by the overpressure introduced by the water in the surface-to-bed fracture and the resulting lake water blister. The difference between the overpressure at the lake input point and the overburden pressure in the remainder of the hydrological system produces a hydraulic potential gradient far greater than that attributed to the ice geometry. We suggest that it is this steep subglacial potential gradient and the short-term separation of the ice and the bed, which allows rapid evacuation of the water from the lake drainage in the form of a turbulent water blister, rather than a network of channels.

Model outputs from *Hewitt* [2013], who tested a catchment designed to emulate the margin of the GrIS, indicate little channel growth from moulin inputs beyond 30 km from the margin. Similar results were found by *Hewitt et al.* [2012] for both steady state and temporally varying subglacial hydrological systems in a Greenland-like domain, with channels essentially absent farther inland than 40 km. *Bougamont et al.* [2014] recently modeled the Russell Glacier catchment using a soft bed configuration, and found surface velocities could be replicated without the presence of subglacial channels. The results from these modeling studies give us confidence in the outputs from our coupled model, which produces similar findings.

### 7.4. Ice Dynamics

Supraglacial lakes have expanded to higher elevations on the GrIS over the last 30 years [*Liang et al.*, 2012; *Howat et al.*, 2013; *Fitzpatrick et al.*, 2014], with greater inland expansion of lakes predicted in a warming climate [*Leeson et al.*, 2015]. There is thus a precedence to establish the dynamic impact of lake drainage events for the interior of the GrIS. Rapid lake drainage events can cause high levels of ice acceleration up to 400% above winter velocities [e.g., *Hoffman et al.*, 2011]. However, this extreme dynamic impact of lake drainage is a short-lived phenomenon lasting less than a day [*Das et al.*, 2008; *Doyle et al.*, 2013; *Tedesco et al.*, 2013]. Our modeling results suggest that it is a turbulent sheet and effective flux through the distributed system that removes the lake drainage water rather than efficient channels as argued by *Howat et al.* [2010], *Sole et al.* [2011], *Bartholomew et al.* [2012], and *Cowton et al.* [2013]. It has been suggested [*Alley et al.*, 2005; *Krawczynski et al.*, 2009; *Gulley et al.*, 2009] that the only plausible mechanism for initiating surface to bed drainage in interior regions of the GrIS is through hydrofracture. This is strengthened by the recent modeling work of *Clason et al.* [2015] who find that moulins are the primary mechanism for water input to the bed at elevations below 1250 m asl, whereas lake drainage events are the most important drainage mechanism above this elevation. These results point toward perhaps the most important role of rapid supraglacial lake drainage: providing the conditions for moulin formation and seasonal access of surface meltwater to the bed. This is particularly the case if low effective pressures persist throughout the melt season, as our model results suggest, possibly allowing ice velocities to be sustained throughout this period (although at a lesser rate than the extreme acceleration measured over the several hours immediately following lake drainage).

Late-summer low velocities identified from remote sensing analysis and GPS studies demonstrate that self-regulation of the Greenland hydrological system can occur as a result of efficient systems causing higher effective pressures [e.g., *Bartholomew et al.*, 2010, 2011b; *Sundal et al.*, 2011]. This is often identified within ∼40–50 km of the ice sheet margin (for example, see studies by *Podrasky et al.* [2012] and *Moon et al.* [2014], examining surface velocity patterns on Jakobshavn Isbræ and Kangiata Nunata Sermia, respectively). On Leverett and Russell Glacier catchments, distinct patterns of low late-summer velocities are also identified up to ∼40 km [*Bartholomew et al.*, 2011b]. In this region there is evidence of annual self-regulation, as discussed by *Sole et al.* [2013] and *van de Wal et al.* [2015], who suggest low winter velocities following particularly warm summers are due to the continued influence of large subglacial channels drawing water from the distributed system for a number of months following cessation of surface water inputs. There is some evidence of this self-regulation at higher elevations on Russell and Leverett catchments [*Sole et al.*, 2013], but this is difficult to accord with channel closure rates under thick ice that would rapidly shut down and repressurize a channelized system following cessation of surface water inputs [*Bartholomaus et al.*, 2007; *Podrasky et al.*, 2012; *Dow et al.*, 2014a], and also our model outputs showing limited channel growth in the region of Lake F.

From the data and modeling evidence, there therefore seems to be a transition region where the subglacial hydrological system develops to an efficient state and causes ice deceleration. It is worth noting here that our definition of interior versus margin divided by the 1000 m asl elevation band is arbitrary and does not necessarily mean that we expect distributed systems to dominate above this level and efficient systems below. Given the limitations of our modeling approach it is not possible for us to determine the exact causes of a transition between an inefficient and efficient system. *Meierbachtol et al.* [2013] suggest that the shallow surface slopes in the interior contribute to a lack of hydraulic potential gradients that would exert a control on the development of efficient drainage systems, and our model slope tests concur with this. It is also possible that the length of time that water has access to the bed during the melt season is a key control for developing an efficient system [*van de Wal et al.*, 2015]. Greater melt accumulation and earlier lake drainage may therefore impact the timing of efficient drainage development in the interior of the GrIS. Yet, if the subglacial hydrological systems in interior regions of the ice sheet remain distributed and inefficient throughout the year, higher water inputs during the summer melt season could cause an increase in annually averaged flow. GPS observations, from a small number of sites in the accumulation area, of a year-on-year increase in ice flow concomitant with an increased extent of melt and supraglacial lakes at high elevations [*Doyle et al.*, 2014; *van de Wal et al.*, 2015] support this assertion. Further data collection and modeling studies would be necessary to confirm that this enhanced flow in the ice sheet interior is due, in part, to a lack of subglacial hydrological development, as our model outputs suggest.

## 8. Conclusion

To investigate the evolution of subglacial hydrology during and following rapid supraglacial lake drainage events in Greenland, we developed a coupled hydrological model that incorporates expansion of a subglacial turbulent water blister concurrent with development of a channelized and distributed drainage system. We forced the model using field data from a lake drainage event located 70 km from the terminus of Russell Glacier in West Greenland. Subsequent development of the subglacial hydrological system was investigated using estimated surface water inputs into the moulin that formed during the lake drainage event. The model was tested using both planar and realistic topography and through a series of sensitivity experiments. Our analysis primarily focused on model outputs of channel growth rates and the impact of this on the effective pressure in the distributed system.

The results of our modeling suggest that the large volumes (>10^6^ m^3^) of water input to the bed of the ice sheet during rapid lake drainage are predominantly evacuated through a turbulent water blister and the distributed drainage system, which is consistent with observations of widespread ice surface uplift during this and previous, similar events [*Das et al.*, 2008; *Doyle et al.*, 2013]. In contrast to some previous studies [*Sole et al.*, 2011; *Cowton et al.*, 2013], we find no support for the development of efficient subglacial channels during rapid drainage. Although the model results indicate that channels do develop from sustained water inputs to moulins following rapid drainage, these channels are still not sufficiently large or efficient to substantially increase the effective pressure in the surrounding distributed system. Our modeling also suggests that variable basal topography, which limits channel growth on reverse slopes, contributes to creating a transient and inefficient drainage system. Sensitivity analyses suggest that gentle surface slopes play a key role in limiting the development of channelized drainage but that the presence of preexisting channels would allow greater channel growth during the melt season. Nevertheless, it is unlikely that such channels would form prior to rapid drainage due to negligible melt inputs to the bed.

The effects of rapid supraglacial lake drainage on ice dynamics are important to constrain as they play a key role in establishing surface-to-bed hydrological connections, especially at high elevations. Our modeling efforts contribute toward this and suggest that a distributed drainage system dominates the subglacial hydrological network in the vicinity of our case study site both during and following rapid lake drainage.
