# Supplementary material for: Modeling of subglacial hydrological development following rapid supraglacial lake drainage
Source: J Geophys Res Earth Surf. 2015 Jun 30;120(6):1127–47. doi: 10.1002/2014JF003333 (PMC4662019; doi:10.1002/2014JF003333)
Supplement: Supplementary file 1 — Text S1, Figure S1, and Tables S1 and S2 [file jgrf0120-1127-sd1.pdf]

Supporting Information for 'Modeling of subglacial hydrological development following rapid supraglacial lake drainage'.

C. F. Dow,[1,2] B. Kulessa,[1] I. C. Rutt,[1] V. C. Tsai,[3] S. Pimentel,[4] S. H. Doyle,[5] D. van As,[6] K. Lindbäck,[7] R. Pettersson,[7] G. A. Jones,[1] and A. Hubbard[5]

Contents of this file

1. Text S1
2. Figure S1
3. Tables S1 to S2

Introduction

In Text S1 of this supplementary material we detail the primary equations for 1) the water blister model by *Tsai and Rice* [2010] and 2) the hydrology component of the ice dynamics flowband model by *Pimentel and Flowers* [2011]. We also describe our filter optimization technique for determining appropriate values for the coupled model sensitivity tests. Figure S1 shows a range of pressure outputs using different exponents for calculating the distributed sheet water pressure. Finally, tables of the model constants

---

Corresponding author: C. F. Dow, Cryospheric Sciences Laboratory, NASA Goddard Space Flight Center, Greenbelt, MD 20771, USA. (christine.f.dow@nasa.gov)

(Table S1) and the Lake F drainage constants (Table S2) are also provided within this document.

Text S1.

1. Water Blister Model

First, we highlight the main equations of the blister model. For clarity, the nomenclature below will be kept primarily the same as in *Tsai and Rice* [2010]. As also given in equation 1 of the main text, the time evolution of the blister radius, L, is

$$\frac{dL}{dt} = C_2\sqrt{\frac{\chi\Delta P}{\rho_w}}\left(\frac{\bar{h}^S}{L}\right)^{2/3}\left(\frac{L}{k}\right)^{1/6}. \tag{1}$$

where C_2 is a constant determined from self-similar analysis (see *Tsai and Rice* [2010]), χ is a friction parameter, ΔP is the water pressure in the connecting vertical crack, ρ_w is the density of water, $\bar{h}^S$ is the average basal opening when applying an elastic plate with clamped edges and uniform loading, and k is the Nikuradse channel roughness height. The volume in the water blister, V_b, is calculated with

$$V_b = C_1\pi L^2\bar{h}^S. \tag{2}$$

where C_1 is a constant. The volume in the vertical englacial crack, V_c, is given by

$$V_c = \frac{2\pi\chi\Delta P_{avg}a^2H_w}{E}, \tag{3}$$

where ΔP_{avg} is the average water pressure in the connecting crack ($\Delta P/2$), a is the horizontal half-length of the englacial crack, H_w is the height of the water in the lake or vertical crack, and E is Young's modulus for ice. The horizontal displacement is related to the elastic opening of the vertical crack, rather than due to melting from frictional

heat generated by water flow, which is not incorporated within this model. Horizontal displacement, u_s, due to pressurized flux in the vertical crack is calculated with

$$u_s(X_0) = \frac{2\chi\Delta P_{avg}a}{E}\left[\sqrt{1+(X_0/a)^2} - \frac{X_0}{a} + \frac{1+\nu}{2}\frac{X_0}{a}\left(1 - \frac{X_0/a}{\sqrt{1+(X_0/a)^2}}\right)\right], \quad (4)$$

where X_0 is the location on the ice surface where the displacement is calculated, and ν is Poisson's ratio. The ice surface uplift, h_s, is given by

$$h_s(X_0) \approx \frac{H^3\bar{h}^S}{\xi\pi^2L^3}\int_{-1}^{1}\frac{\hat{w}(\hat{x})\,d\hat{x}}{[((\frac{x}{L}) - (\frac{X_0}{L}))^2 + (\frac{H}{L}^2)]^2}, \quad (5)$$

where ξ is a is a constant coefficient of proportionality adjusting homogeneous crack opening calculations (w) to a bimaterial case ($\bar{h}^S$), H is the ice thickness and x the horizontal distance along the basal crack. Variables with hats represent non-dimensionalized versions of the respective unhatted parameters.

2. Hydraulic Flowline Model

We now provide additional equations from the hydrology component of the *Pimentel and Flowers* [2011] flowband ice dynamics model; the flux and channel evolution equations are included in the main text. The gradient of hydraulic potential ($\partial\psi/\partial x$) is calculated following *Shreve* [1972] with

$$\frac{\partial \psi}{\partial x} = \Delta P_w + \rho_w g \Delta b, \tag{6}$$

where b is the bed elevation, ρ_w is the density of water, P_w is the water pressure for either the channel (P_w^c) or the sheet (P_w^{sh}) depending on the application of the equation, and g is the gravitational acceleration. The time dependence of channel water pressure is determined by

$$\frac{\partial P_w^c}{\partial t} = -\frac{1}{\gamma S}\left[\frac{\partial S}{\partial t} + \frac{\partial Q^c}{\partial x} + \frac{Q^c}{\rho_w L_f}\left(\frac{\partial \psi}{\partial x} - c_p \rho_w C_w \frac{\partial P_w^c}{\partial x}\right) - d_c\left(\dot{b}^c + \phi^{sh:c}\right)\right], \tag{7}$$

where γ is an artificial parameter to overcome model stiffness [see *Clarke*, 2003], S is the channel cross-sectional area, Q^c is the channel discharge, L_f is the latent heat of fusion, c_p is the specific heat capacity of water, C_w is the Clausius-Clapeyron gradient, d_c is the lateral channel spacing within the domain, $\dot{b}^c$ is the water source term to the channel and $\phi^{sh:c}$ is the exchange between the channel and the sheet, following the approach of *Flowers* [2008]:

$$\phi^{sh:c} = X^{sh:c}\frac{K h^{sh:c}}{\rho_w g d_c^2}(P_w^{sh} - P_w^c), \tag{8}$$

where $X^{sh:c}$ is an exchange coefficient, K is the hydraulic conductivity, and $h^{sh:c}$ is the water sheet thickness, with the pressure used in the thickness calculation determined by the maximum pressure for that node in either the channel or the sheet. The temporal change in water sheet thickness is given by

$$\frac{\partial h^{sh}}{\partial t} = -\frac{\partial q^{sh}}{\partial x} + \frac{Q_G}{\rho_i L_f} + \dot{b}^{sh} - \phi^{sh:c}. \tag{9}$$

where q^{sh} is the vertically-integrated water flux in the sheet, Q_G is the geothermal heat flux, ρ_i is the density of ice, and $\dot{b}^{sh}$ is the source term for the distributed sheet. The sheet water pressure is calculated with

$$P_w^{sh} = P_i \left(\frac{h^{sh}}{h_c^{sh}} \right)^{\beta}, \tag{10}$$

where h_c^{sh} is the critical water sheet thickness (when water pressure is at overburden), P_i is the ice overburden pressure, and β is a parameter determined by the equation application. *Flowers and Clarke* [2002] empirically calculated P_w^{sh}, for Trapridge Glacier using β=7/2 and this relationship was also used in the *Pimentel and Flowers* [2011] model. From the Lake F GPS measurements and from the blister model, it appears that subglacial water can temporarily exist at a thickness of tens of centimeters at the ice-bed interface, coincident with water pressures above overburden. Using Eq. 10 to examine the relationship between water pressure and water sheet thickness produces the following results: with β=7/2, if the blister water thickness is 0.3 m and the critical sheet water thickness (for water at overburden) is 0.15 m, the water pressure would be $11 \times P_i$. Given a maximum uplift of ~0.8 m at the case-study lake site, if this directly translates to water thickness at the bed, the pressure according to Eq. 10 would be $350 \times P_i$. Figure S1a plots the range of water pressures output for our sensitivity test critical water thickness values using this value of beta. Given that water pressures are expected to fluctuate around $1 \times P_i$ as an upper bound, these calculated values are clearly unrealistic and β=7/2 is therefore inappropriate for periods when water pressures are above overburden. As a result, we apply Eq. 10 so that when $P_w^{sh} > P_i$, $\beta = 1/13$ and when $P_w^{sh} \leq P_i$, $\beta = 7/2$. This value of beta for times of overpressure is estimated using the relationship between the calculated blister

water thickness at the input point and the known pressure when the water is at the ice surface (assuming a fully-vertical englacial crack). Using this equation, if the blister water thickness is 0.3 m and the critical water thickness (for water at overburden) is 0.15 m, the pressure would be $1.05 \times P_i$; and for a blister thickness of 0.8 m, $P_w^{sh} = 1.14 \times P_i$. The appropriateness of this value of beta is demonstrated in Figure S1b and, without in situ borehole data of the water pressures at Lake F, is our best estimate for the pressure conditions at levels above overburden.

3. Filter Optimization Technique

Within the water blister model, different model set-ups are possible based on the initial conditions of the blister radius, L, and the ratio between L and the horizontal extent of the connecting vertical crack, a. The blister model was therefore tested with initial crack radii of 10, 20 and 50 m (as without an initial crack, the fracture equations could not be applied). Within each of these initial blister conditions, a/L was varied at 0.85, 0.95 and 1. For a/L <0.85, the model outputs are sufficiently incomparable to the case-study surface data that they were not further tested.

If these nine L and a/L blister sensitivity tests were included in every parameter sensitivity test it would result in a total of >1000 full model outputs. The time required to run all these tests is beyond the scope of this study. As an alternative, a filter optimization technique was applied to the planar model geometry sensitivity runs, with an outlet pressure value of P_i. For these optimization tests, the Stage I spin-up model was run followed by the Stage II linked model for a maximum of one model hour to obtain the blister model outputs. In total, the nine L and a/L blister sensitivity tests were then run for the sensitivity parameters shown in Table 2 of the main text. The outputs from the blister

model were compared with the data from the case-study site, including the lake lowering rate and the GPS station uplift and horizontal motion records. The lake lowering rate was used as the primary control for determining the most appropriate internal parameter choice of initial L and a/L, as the GPS records might be contaminated with uplift due to ice block fracturing rather than uplift only as a result of water blister expansion. In order to compare the blister model lake level outputs (H_w) with the recorded Lake F lowering level, a trapezoidal integration method was applied to both. The three (of nine) model H_w outputs that most closely matched the area under the curve of the Lake F water level were weighted as the primary parameters. In these tests, the integrated water level value was often the same or at least similar for three of the nine tests, and as a result, a secondary filter optimization technique was necessary. Of these three parameters, the best combination of L and a/L for the tested sensitivity parameter was therefore chosen by comparing the blister model horizontal displacement outputs with the horizontal motion records from GPS_NW. The primary parameter that had the closest temporal match for 0.1 m of horizontal motion when compared to the GPS_NW record was chosen as the final blister model initial condition.

It was found that, for the majority of these optimization tests, an initial blister radius of 50 m was appropriate. However, for a Young's modulus value of 3 GPa, the initial blister size of 10 m was a better match; for E=8.84 GPa the blister size of 20 m was most appropriate. Also, with a critical water thickness >0.15 m, L=20 m was the closest match to the surface data. In all model runs, a/L=0.85 was the most appropriate match. In the sensitivity tests, a/L is constantly set at 0.85 but the initial L length is varied depending on the optimization results described above.

References

Clarke, G. K. C. (2003), Hydraulics of subglacial outburst floods: new insights from the Spring-Hutter formulation, *Journal of Glaciology*, *49*(165), 299–314.

Flowers, G. E. (2008), Subglacial modulation of the hydrograph from glacierized basins, *Hydrological Processes*, *22*(19), 3903–3918.

Flowers, G. E., and G. K. C. Clarke (2002), A multicomponent coupled model of glacier hydrology: 1. Theory and synthetic examples, *Journal of Geophysical Research*, *107*(B11), 2287.

Pimentel, S., and G. E. Flowers (2011), A numerical study of hydrologically driven glacier dynamics and subglacial flooding, *Proceedings of the Royal Society A: Mathematical, Physical and Engineering Science*, *467*(2126), 537–558.

Shreve, R. L. (1972), Movement of water in glaciers, *Journal Of Glaciology*, *11*(62), 205–214.

Tsai, V. C., and J. R. Rice (2010), A model for turbulent hydraulic fracture and application to crack propagation at glacier beds, *Journal of Geophysical Research*, *115*(F3), F03,007.

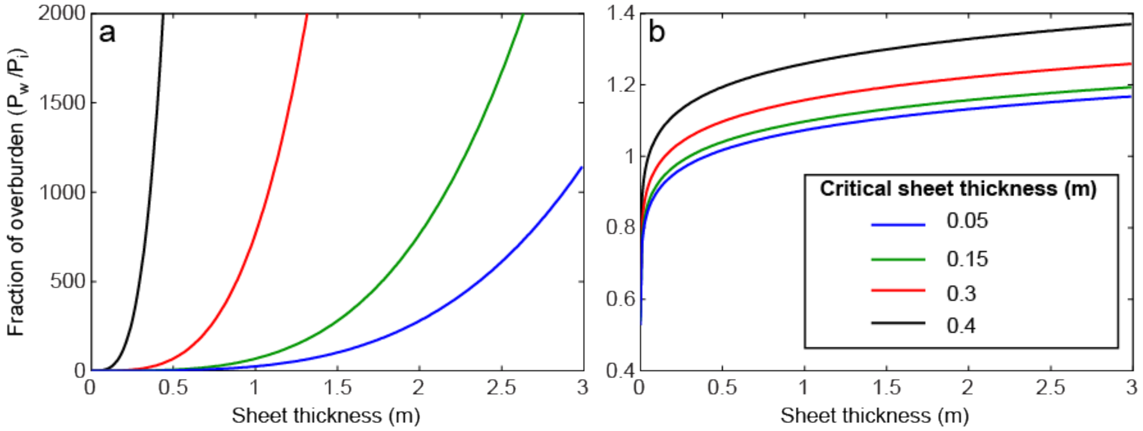

Figure S1. Plots of water pressure (as a percentage of overburden) for the range of critical water thickness values used in our sensitivity tests when applying equation 10 with a) $\beta = 7/2$ and b) $\beta = 1/13$.

Table S1. Coupled hydrological model constants.

Symbol	Description	Value	Units
C_1	Uplift scaling factor	0.592	dimensionless
C_2	Velocity scaling factor	3.571	dimensionless
c_p	Specific heat capacity of water	4.22×10^3	J kg^{-1} K^{-1}
C_w	Clausius-Clapeyron gradient	9.8×10^{-8}	K Pa^{-1}
f_0	Darcy-Weisbach friction parameter	0.143	s m$^{-1/3}$
g	Acceleration due to gravity	9.81	m s^{-2}
L_f	Latent heat of fusion	3.35×10^5	J kg^{-1}
n	Glen's flow law exponent	3	dimensionless
Q_G	Geothermal heat flux	0.07	W m^{-2}
γ	Numerical compressibility parameter	10^{-9}	Pa^{-1}
η	Dynamic viscosity of water	1.78×10^{-3}	Pa s^{-1}
ν	Poisson's ratio	0.3	dimensionless
ξ	Ratio of h to w	0.55	dimensionless
ρ_i	Density of ice	910	kg m^{-3}
ρ_w	Density of water	1000	kg m^{-3}

Table S2. Lake F model parameters.

Symbol	Description	Value	Units
A_0	Lake surface area	2.6×10^6	m^2
d_n	Number of channels	1	dimensionless
d_c	Channel spacing	1500	m
V_0	Lake volume	7.1×10^6	m^3
W	Flowline width	1500	m
δx	Grid-cell size	100	m
